# Notes on Parasites

**Published:** 1892-07

**Authors:** M. Francis

**Affiliations:** Veterinarian; Texas Experiment Station


### NOTES ON PARASITES.

By M. Francis, Veterinarian.

In the March issue of the Journal and of the *Review* there appeared a note by Dr. Stiles, on the large liver fluke of cattle (D. Texanicum), which will bear a word of explanation.

Dr Stiles stated on page 732 of the *Review* that "this parasite, however, has been known for *some time* (italics mine) to the Bureau of Animal Industry." I beg to state that it was known to *others* for some time. In the Report of the Arkansas Exp't Station, for 1889, Dr. Dinwiddie gave an account of this parasite under the name of F. Hepatica. Although Dr. Dinwiddie's report is almost ruined by this error, the cut that appears there and the description that accompanies it, is characteristic of the parasite in question.

It has been known to still others for "*some time.*" When Dr. Cooper Curtice visited me in October, 1890, we had a conversation on the subject, when examining this parasite, in which he stated that he had collected the same in Colorado some time before that.

It seems to me in discussing such a subject, in which question of fact and of priority are involved, Dr. Stiles should have the fairness to mention the work of others. One word more. Dr. Hassell wrote me May 14, 1891, for "information concerning Fluke disease in farm stock, due to Fasciola Hepatica, or any other Trematode parasite." I wrote to Dr. Hassell at once, giving him a synopsis of bulletin eighteen of Texas Exp't Station, then in MSS.

Dr. Hassell immediately on receipt of my letter sent to the *Review* a description of the large fluke *as a discovery*. So hastily was this gotten up that Dr. Liautard did not publish it, but wrote Dr. Hassell for a drawing. This drawing Dr. Hassell constructed from mutilated specimens in the collection of the Bureau of Animal Industry, and both appear in the *Review* for July, 1891. I find on further investigation that the statement made, viz: "I think they die in these cysts" to be often correct, as I have recently collected a number in this condition. They become pale, straw colored, the digestive tract becomes empty, and they gradually disintegrate.

2. It may be of interest to state the occurrence of a species of Argas in this portion of the country. Last July a physician of

San Marcos, Tex., sent us specimens of this that were killing chickens. He stated that kerosene proved an efficient remedy for their destruction. *Amblyomma unipunctata, P.* "The Lone Star Tick" has been very abundant the present season. I have collected it from horse, mule, ox, dog and cat. It is especially abundant in the ears of dogs and genitals of horses. This tick, when in the ears of horses or mules, makes it almost impossible to put on the bridle—only a few are necessary to make a mule really dangerous to attempt to bridle.

3. Dr. Curtice makes a slight error on page 226 in speaking of the "Screw Worm," Lucilia Macellaria, as occurring in the *Fourth* Stomach of a calf. It should read in the *First Stomach*, *Rumen* or Paunch.

4. These Screw Worms frequently are found in the ears of the horse, and if not destroyed at once, often cause a very conspicous deformity of the Concha, which cannot be remedied, called "gotch ear."

TEXAS EXPERIMENT STATION, June 7, 1892.

